# Positional weight matrices have sufficient prediction power for analysis of noncoding variants

**DOI:** 10.12688/f1000research.75471.1

**Published:** 2022-01-12

**Authors:** Alexandr Boytsov, Sergey Abramov, Vsevolod J. Makeev, Ivan V. Kulakovskiy

**Affiliations:** 1 Vavilov Institute of General Genetics, Russian Academy of Sciences, Moscow, 119991, Russian Federation; 2 Moscow Institute of Physics and Technology, Dolgoprudny, 141700, Russian Federation; 3 Institute of Protein Research, Russian Academy of Sciences, Pushchino, 142290, Russian Federation

**Keywords:** Transcriptional regulation, rSNP, TF-DNA binding, SNP-SELEX, PWM, PSSM

## Abstract

The commonly accepted model to quantify the specificity of transcription factor binding to DNA is the position weight matrix, also called the position-specific scoring matrix. Position weight matrices are used in thousands of projects and computational tools in regulatory genomics, including prediction of the regulatory potential of single-nucleotide variants. Yet, recently Yan *et al*. presented new experimental method for analysis of regulatory variants and, based on its results, reported that "the position weight matrices of most transcription factors lack sufficient predictive power". Here, we re-analyze the rich experimental dataset obtained by Yan *et al*. and show that appropriately selected position weight matrices in fact can successfully quantify transcription factor binding to alternative alleles.

## Introduction

Gene regulatory regions constitute an important part of non-coding DNA which defines both the global development program of a mammal and individual traits of a particular organism. Specific recognition of DNA sites by transcription factors (TFs) provides the gear system linking individual genomic variants to phenotypes. ^1^ The commonly accepted model to quantify the specificity of transcription factor binding to various DNA sites is the position weight matrix (PWM), which specifies additive contributions of individual nucleotides to the protein-DNA binding energy. ^2^ Recently Yan *et al*. ^3^ reported that “the position weight matrices of most transcription factors lack sufficient predictive power” for assessment of regulatory variants identified with a new experimental method (SNP-SELEX). This finding could be devastating for a vast array of research projects and software tools which use PWMs for prediction of the regulatory potential of single-nucleotide variants. ^4^^–^^7^ Here, we re-analyze the dataset of Yan et al. and argue that the transcription factor binding to alternative alleles detected by SNP-SELEX can be described quantitatively by carefully selected PWMs.

To rehabilitate PWMs as predictive models of TF-DNA binding, we used the CIS-BP (Catalog of Inferred Sequence Binding Preferences) collection ^8^^,^^9^ of pre-made matrices instead of PWMs of Yin *et al*. ^10^ For each TF, we additionally considered PWMs for related proteins sharing similar DNA binding domains as the benchmarking study of Ambrosini *et al*. ^2^ demonstrated that PWMs of related TFs often outperform those for the target TF.

**Figure 1.**
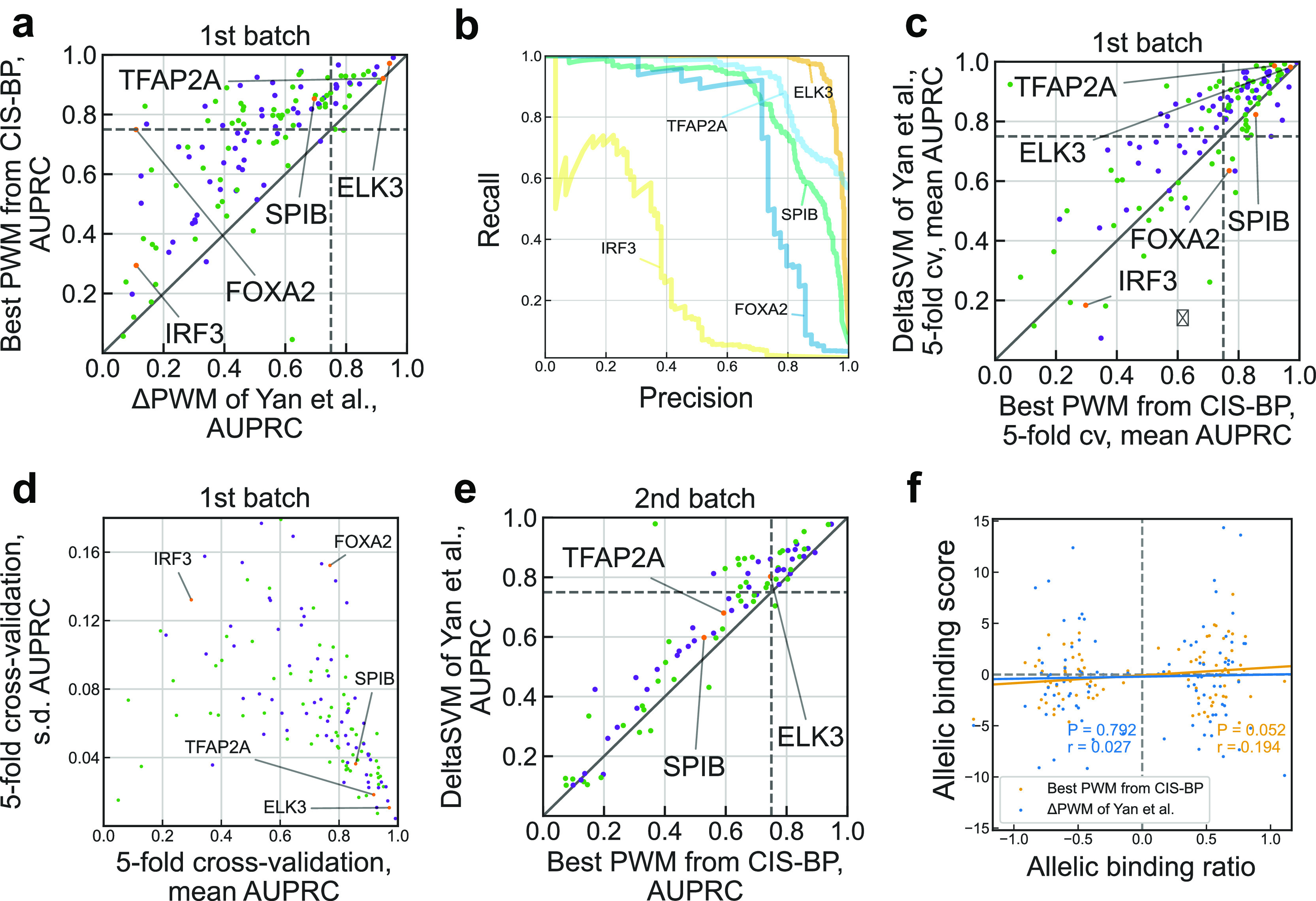
Re-evaluation of position weight matrices with the SNP-SELEX data. a. Comparison of performance of Yan *et al*. ΔPWM (x-axis) and best CIS-BP position weight matrices (PWMs) in predicting preferential binding SNPs in the 1st batch on the SNP-SELEX data. Each point denotes one of 129 TFs, violet and green points denote inferred and direct PWMs, respectively (see the Methods). Both axes show area under the precision-recall curve (AUPRC) values. Transcription factors (TFs) shown in Fig. 2b of Yan *et al*. are highlighted in orange and labeled. Dashed lines denote AUPRC of 0.75. b. Examples of the precision-recall curves showing performance of different PWM models in predicting preferential binding SNPs (single-nucleotide polymorphisms) as in Fig. 2b of Yan *et al*. c. Comparison of performance of deltaSVM (y-axis) and best CIS-BP PWMs (x-axis) in predicting preferential binding SNPs identified in the 1st batch of SNP-SELEX. Each point denotes one of 129 TFs, violet and green points denote inferred and direct PWMs, respectively. Both axes show mean AUPRC values obtained by 5-fold cross-validation (cv). Dashed lines denote AUPRC of 0.75. d. Variance of performance of CIS-BP PWMs (x-axis: mean AUPRC, y-axis: s.d.) in 5-fold cross-validation using the complete data of the 1st batch of SNP-SELEX. Each point denotes one of 129 TFs, violet and green points denote inferred and direct PWMs, respectively. e. Comparison of performance of deltaSVM (y-axis) and best CIS-BP PWMs (x-axis) in predicting preferential binding SNPs identified in the 2nd batch of SNP-SELEX. Each point denotes one of 87 TFs, violet and green points denote inferred and direct PWMs, respectively. Both axes show AUPRC values. Dashed lines denote AUPRC of 0.75. f. Correlation of allelic biases of DNA binding detected from ChIP-Seq experiments in HepG2 cells by Yan *et al*. and those predicted by ΔPWM of Yan *et al*. (blue) and best CIS-BP PWMs (orange). Pearson correlation coefficient ( *r*) and the respective *P*-value are shown. The allelic binding ratio is computed as in Yan *et al*.; 101 transcription factor-SNP pairs involving 68 unique SNPs and 6 transcription factors (ATF2, FOXA2, HLF, MAFG, YBX1, and FOXA1) are shown.

With the 1st batch of SNP-SELEX data of Yan *et al*., we found that for more than a half (72 of 129) of transcription factors the best PWMs achieve reliable predictions (with the same criterion as in Yan *et al*. requiring area under the precision-recall curve (AUPRC) > 0.75, see Figure 1a). This is 3 times more transcription factors with reliable PWM predictions than reported in Yan *et al*. We obtained good predictions in some cases reported as markedly underperforming such as FOXA2 (compare Figure 1b with Fig. 2b of Yan *et al*.). Furthermore, the achieved performance allows PWMs to compete with and, for 34 transcription factors, outperform advanced models of deltaSVM, recommended by Yan *et al*. as a substitution for PWMs ( Figure 1c). To ensure the reliability of these results, we performed 5-fold cross-validation, which showed that models reaching higher AUPRC simultaneously had a lower variance in prediction quality across individual folds ( Figure 1d). Furthermore, we tested the PWMs on the independent 2nd batch data ( Figure 1e, compare with Fig. 3d of Yan *et al*.), and it also showed competitive albeit lower performance, with 36 of 124 transcription factors passing 0.75 AUPRC. Finally, we tested if the PWM predictions agree with the allelic binding ratios and found a small but marginally significant correlation ( Figure 1f, *r* = 0.194, *P* = 0.052) for 101 SNPs tested in Yan *et al*. and reaching *r* = 0.235 ( *P* = 0.047) for a subset of 72 SNPs with significant PWM hits (motif *P*-value < 0.005), in contrast to almost zero correlation for ΔPWM reported in Yan *et al*.

Summing up, our results do not compromise the high performance of deltaSVM, ^11^ used by Yan *et al*. as an advanced substitution of position weight matrices (PWMs). However, properly selected PWMs achieve performance that is very close and in some cases even better than that of deltaSVM. Despite the simplicity of the PWM model, its construction is not trivial and its success depends both on the motif discovery algorithm and reliability of the training data. In our case, almost half of the best PWMs were derived from related TFs, including 8 cases of PWMs based on experimental data from other species. The experiments used to obtain the best PWMs were also of different types, including ChIP-Seq, protein-binding microarrays, and SMiLE-Seq data, see *Extended data,* Supplementary Table S1. ^12^ Thus, it is important to consider various sources of PWMs and select those the most suitable by proper benchmarking. In the context of applying PWMs to analyze regulatory variants, SNP-SELEX of Yan *et al*. provides rich, unique, and practically useful data. Advanced multiparametric and alignment-free approaches such as deltaSVM appear very likely to shape the oncoming future of transcription factor binding site models, but today PWMs still deliver a solid standard in representation and bioinformatics analysis of the transcription factor binding sites, including assessment of the functional impact of single nucleotide variants in gene regulatory regions.

## Methods

### PWMs used in the study

The starting set of position frequency matrices was extracted from TF_Information_all_motifs.txt of CIS-BP 2.0 that includes models derived from direct experimental data for each TF and models that can be inferred given the TF family-specific threshold on DNA-binding domain similarity, see Ref. 10, referred to in Figure 1 caption as ‘direct’ and ‘inferred’ PWMs. All position frequency matrices were converted to log-odds PWMs as in Ref. 13 with an arbitrarily selected word count of 100, a pseudocount of 1, and uniform background nucleotide probabilities. For each TF, the set of PWMs was additionally extended by considering related TFs, i.e. PWMs for all ETV* TFs were added to the ETV1 PWM set, all FOX* (Forkhead box) PWMs were added to the FOXA2 PWM set, etc. (e.g. YY1 and YY2 PWM sets were identical). This procedure was not performed for ZNF* (zinc finger) TFs as these TFs can recognize very dissimilar motifs and thus additional PWMs of other ZNFs would unlikely provide any benefit. The resulting set contained a median of 32 PWMs per TF although the overall distribution was non-uniform e.g. only 2 PWMs for ZNF396 and over a thousand for FOXA2, see *Extended data,* Supplementary Table S1. Upon assessment with the SNP-SELEX data, there was no correlation between the prediction performance (AUPRC) and the number of tested PWMs per TF ( *r* = −0.07, *P* = 0.425).

### Determination of transcription factor binding preference using PWMs

To assess with a particular PWM whether an SNV affects transcription factor binding, we used PERFECTOS-APE ^5^ that estimates the log-fold change of motif P-values computed for best PWM hits detected among sites overlapping the first and the second of two alternative alleles. To use the prediction as a binary classifier, we treated the cases with *P* > 0.005 at both alleles as predicted negatives and used the log-fold change as the prediction score in the remaining cases. The auc function of the sklearn.metrics Python package was used to estimate the area under the precision-recall curve (AUPRC).

### Estimating PWM performance with SNP-SELEX data

To provide a fair assessment, we mimicked the benchmarking protocol of Yan *et al*. Particularly, true positives and true negatives were selected from the SNP-SELEX data as follows. 1st batch data positives: pbs *P*-value < 0.01 and obs *P*-value < 0.05; negatives: pbs *P*-value > 0.5 and obs *P*-value < 0.05. 2nd batch data positives: pbs *P*-value < 0.01, negatives: pbs *P*-value > 0.5. For each TF, we tested each PWM from its PWM set. For each TF, the PWM reaching the highest AUPRC on the 1st batch data was selected for evaluation against the best ΔPWM on the 1st batch ( Figure 1a) and against deltaSVM on the 2nd batch of SNP-SELEX data ( Figure 1e). Performance estimates for deltaSVM models (used in Figure 1c,e) were extracted from Supplementary Table S7 of Yan *et al*. Performance estimates of ΔPWM (used in Figure 1a) were kindly shared on our request by the authors. ^3^

### Applying PWMs for analysis of allele-specific binding

The data on allelic binding ratios at individual SNPs and respective ΔPWM predictions of Yan *et al*. ( Figure 1f, compare to Fig. 2d of Yan *et al*.) were kindly shared on our request by the authors. The data included 193 TF-SNP pairs demonstrating allelic imbalance with 101 of 193 pairs annotated with the ΔPWM predictions. For these SNPs, we obtained PWM predictions with the same protocol as for the SNP-SELEX data using the best PWMs selected with the 1st batch of the SNP-SELEX data.

## Data availability

### Source data

Original data on preferential binding SNPs as well as ΔPWM and deltaSVM predictions are provided in the supplementary materials section of the Yan *et al*. paper. ^3^

CISBP Human PWMs collection was extracted from CIS-BP 2.0. ^8^^,^^9^

### Extended data

Figshare: PWM-evaluation-using-SNP-SELEX, https://doi.org/10.6084/m9.figshare.16906789.v1. ^12^

This project contains the following extended data:

• **Supplementary table S1** (Overview of PWMs and their performance in recognizing SNPs affecting transcription factor binding in SNP-SELEX data.)

Data are available under the terms of the Creative Commons Zero “No rights reserved” data waiver (CC0 1.0 Public domain dedication).

## Acknowledgements

This study was supported by Russian Science Foundation grant 20-74-10075 to IVK.

## References

[1] Wasserman WW Sandelin A : Applied bioinformatics for the identification of regulatory elements. *Nat. Rev. Genet.* 2004;5:276–287. 10.1038/nrg1315 15131651

[2] Ambrosini G : Insights gained from a comprehensive all-against-all transcription factor binding motif benchmarking study. *Genome Biol.* 2020;21:114. 10.1186/s13059-020-01996-3 32393327 PMC7212583

[3] Yan J : Systematic analysis of binding of transcription factors to noncoding variants. *Nature* 2021;591:147–151. 10.1038/s41586-021-03211-0 33505025 PMC9367673

[4] Macintyre G Bailey J Haviv I : is-rSNP: a novel technique for in silico regulatory SNP detection. *Bioinformatics* 2010;26:i524–i530. 10.1093/bioinformatics/btq378 20823317 PMC2935445

[5] Vorontsov IE Kulakovskiy IV Khimulya G : PERFECTOS-APE - Predicting Regulatory Functional Effect of SNPs by Approximate P-value Estimation. *Proceedings of the International Conference on Bioinformatics Models, Methods and Algorithms 102–108 (SCITEPRESS - Science and and Technology Publications* 2015. 10.5220/0005189301020108

[6] Coetzee SG Coetzee GA Hazelett DJ : motifbreakR: an R/Bioconductor package for predicting variant effects at transcription factor binding sites. *Bioinformatics* 2015;31:btv470–bt3849. 10.1093/bioinformatics/btv470 26272984 PMC4653394

[7] Deplancke B Alpern D Gardeux V : The Genetics of Transcription Factor DNA Binding Variation. *Cell* 2016;166:538–554. 10.1016/j.cell.2016.07.012 27471964

[8] Lambert SA : The Human Transcription Factors. *Cell* 2018;172:650–665. 10.1016/j.cell.2018.01.029 29425488 PMC12908702

[9] Weirauch MT : Determination and Inference of Eukaryotic Transcription Factor Sequence Specificity. *Cell* 2014;158:1431–1443. 10.1016/j.cell.2014.08.009 25215497 PMC4163041

[10] Yin Y : Impact of cytosine methylation on DNA binding specificities of human transcription factors. *Science* 2017;356:eaaj2239. 10.1126/science.aaj2239 28473536 PMC8009048

[11] Lee D : A method to predict the impact of regulatory variants from DNA sequence. *Nat. Genet.* 2015;47:955–961. 10.1038/ng.3331 26075791 PMC4520745

[12] Abramov S : PWM evaluation using SNP-SELEX. Online resource. 160773 Bytes 2021. 10.6084/M9.FIGSHARE.16906789.V1

[13] Lifanov AP Makeev VJ Nazina AG : Homotypic Regulatory Clusters in Drosophila. *Genome Res.* 2003;13:579–588. 10.1101/gr.668403 12670999 PMC430164

