# Positional weight matrices have sufficient prediction power for analysis of noncoding variants

**DOI:** 10.12688/f1000research.75471.3

**Published:** 2022-07-04

**Authors:** Alexandr Boytsov, Sergey Abramov, Vsevolod J. Makeev, Ivan V. Kulakovskiy

**Affiliations:** 1 Vavilov Institute of General Genetics, Russian Academy of Sciences, Moscow, 119991, Russian Federation; 2 Moscow Institute of Physics and Technology, Dolgoprudny, 141700, Russian Federation; 3 Institute of Protein Research, Russian Academy of Sciences, Pushchino, 142290, Russian Federation

**Keywords:** Transcriptional regulation, rSNP, TF-DNA binding, SNP-SELEX, PWM, PSSM

## Abstract

The position weight matrix, also called the position-specific scoring matrix, is the commonly accepted model to quantify the specificity of transcription factor binding to DNA. Position weight matrices are used in thousands of projects and software tools in regulatory genomics, including computational prediction of the regulatory impact of single-nucleotide variants. Yet, recently Yan et al. reported that "the position weight matrices of most transcription factors lack sufficient predictive power" if applied to the analysis of regulatory variants studied with a newly developed experimental method, SNP-SELEX. Here, we re-analyze the rich experimental dataset obtained by Yan et al. and show that appropriately selected position weight matrices in fact can adequately quantify transcription factor binding to alternative alleles.

## Introduction

Gene regulatory regions constitute an important part of non-coding DNA which defines both the global development program of a mammal and individual traits of a particular organism. Specific recognition of DNA sites by transcription factors (TFs) provides the gear system linking individual genomic variants to phenotypes. ^1^ The commonly accepted model to quantify the specificity of transcription factor binding to various DNA sites is the position weight matrix (PWM), which specifies additive contributions of individual nucleotides to the protein-DNA binding energy. ^2^ ^,^ ^3^ Recently Yan *et al*. ^4^ presented a powerful high-throughput experimental technique, SNP-SELEX, which allows measuring differential TF binding to alternative alleles *in vitro.* Yan *et al.* used the experimental data they had obtained for many TFs to assess the performance of PWM in predicting differential TF binding to alternative alleles and compare it to that of deltaSVM, a more complex method based on machine learning. As a result, they reported that in this setting “the position weight matrices of most transcription factors lack sufficient predictive power”. Keeping in mind that PWMs are extensively used for prediction of the regulatory potential of single-nucleotide variants ^5^ ^–^ ^8^ the finding of Yan *et al.* could be devastating for a vast array of research projects and software tools.

Yan *et al.* tend to explain the poor performance of PWMs by model limitations, primarily, arising from the oversimplistic assumption that nucleotides occupying different positions in the binding site provide independent contributions to the binding energy. Here we re-analyze the dataset of Yan *et al.* and argue that the poor PWM performance in predicting differential transcription factor binding to alternative alleles detected by SNP-SELEX is to a major extent explained not by the principle limitations of PWM as a mathematical construction but rather by particular inadequate PWMs for TFs under study. We show that the careful selection of PWMs of many TFs from a public database quantitatively explains the differential TF binding to allelic variants with reliability comparable to deltaSVM.

## Results

To re-assess PWM performance, we used PWMs stored in the CIS-BP database, ^9^ ^,^ ^10^ which contains PWMs constructed from data obtained with different experimental techniques for thousands of TFs for different species. With the objective of selecting the PWM appropriate for quantifying differential allele binding of a TF, for each of 129 TFs assessed in Yan *et al.* we extracted an extended set of candidate PWMs, with a median of 32 PWMs per TF. The overall distribution was non-uniform e.g. there were only 2 candidate PWMs for ZNF396 and over a thousand for FOXA2, see *Extended data,* Supplementary Table S1.

Through cross-validation on the 1st batch of SNP-SELEX data following the strategy of Yan *et al*., we selected the best PWM ^CIS-BP^ for each TF (see “Selecting the best PWMs and estimating PWM performance with SNP-SELEX data” in the Methods). There was no correlation between the prediction performance (area under precision-recall curve, AUPRC) and the number of tested PWMs per TF ( *r* = −0.07, *P* = 0.425). Many of the best-performing PWMs were originally constructed from the data related not to the target TF but to other TFs sharing the same DNA-binding domain as the TF of interest. Some PWMs were based upon the TF binding data from different species. We denote by ΔPWM ^CIS-BP^ the difference of the allelic scores predicted with PWM ^CIS-BP^ and by ΔPWM ^Mult^ the results of PWMs obtained from HT-SELEX data using the multinomial algorithm ^11^ and assessed by Yan *et al.* ^4^

**Figure 1.**
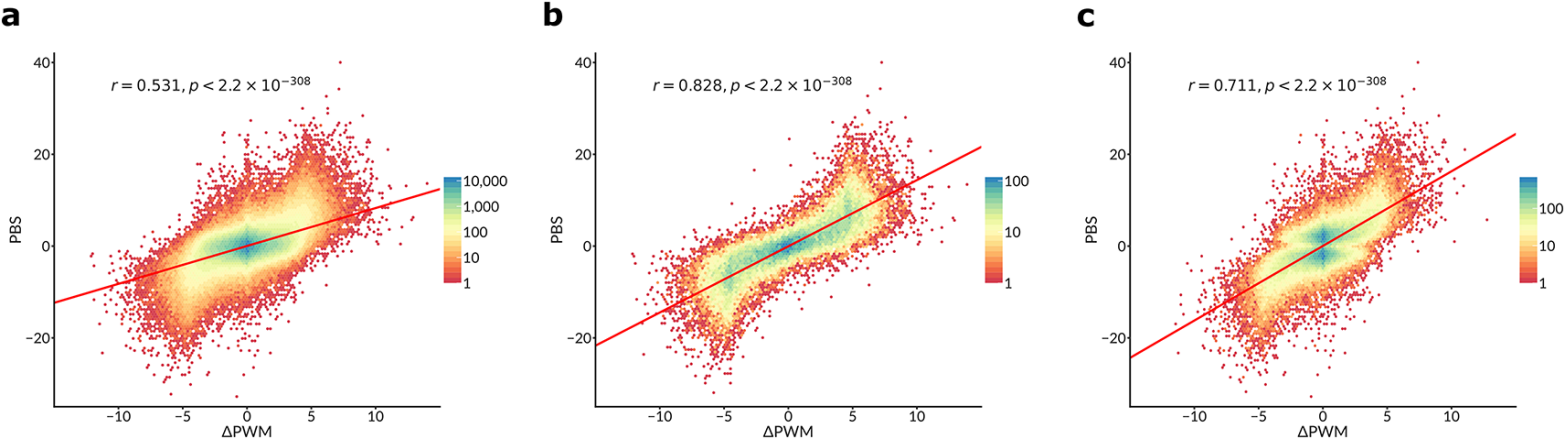
PWM predictions of differential TF binding to SNPs correlate with SNP-SELEX estimates. Hex-plots of PBS scores (Y-axis) vs ΔPWM ^CIS-BP^ predictions (X-axis) for different sets of SNPs, analogous to Figure 2a and Figure 3a in Yan *et al.*, with PCC and two-sided t-test *P*-values displayed. a. Complete set of 816594 TF-SNP pairs for 129 TFs that were used to compare the performance of deltaSVM vs ΔPWM in Yan *et al.* b. A subset of 35837 TF-SNP pairs overlapping strong PWM hits ( *P* < 10 ^-4^ for a stronger bound allele). c. A subset of 84962 TF-SNP pairs obtained by considering only the TF with the highest PBS score for each SNP (as in Figure 3a of Yan *et al*).

Yan *et al.* provide the experimental differential binding scores for each SNP (the “pbSNP” scores). We compared these scores with ΔPWM ^CIS-BP^ predictions. For a complete set of 816594 TF-SNP pairs ( Figure 1a), the Pearson correlation coefficient was comparable to that observed by Yan *et al.* (0.531 vs 0.534 in Yan *et al.*, see their Figure 2a). Yet, if only SNPs with a strong predicted binding are included ( *P* < 10 ^-4^ for the PWM score of a stronger bound allele) then a much higher correlation (r ~ 0.828) is achieved, see Figure 1b. Finally, if only the most strongly bound TF is considered for each SNP (as in Figure 3a of Yan *et al.*), the respective correlation reaches 0.711, comparable to -0.777 reported for deltaSVM in Yan *et al.* (compare to their Figure 1c).

To assess ΔPWM ^CIS-BP^ performance at varying binding affinity ranges we, similarly to Yan *et al.*, categorized the SNPs into five quantiles based on their observed affinities (“OBS” scores) and assessed the performance of ΔPWM ^CIS-BP^ separately for each quantile. For all quantiles but the lowest (the weakest bound sites) ΔPWM ^CIS-BP^ outperformed ΔPWM ^Mult^ of Yan *et al.* Notably, the performance of ΔPWM ^CIS-BP^ was especially high for the middle quantiles and for the highest quantile was on par with deltaSVM (see Supplementary Figure S1, compare with Extended data Figure 7 in Yan *et al.*). Particularly, for strongly bound SNPs from high quantiles in the first SNP-SELEX batch, ΔPWM ^CIS-BP^ did not display any TFs with a very small AUPRC (i.e. prediction failures), the other metric for which deltaSVM dramatically outperformed ΔPWM ^Mult^.

**Figure 2.**
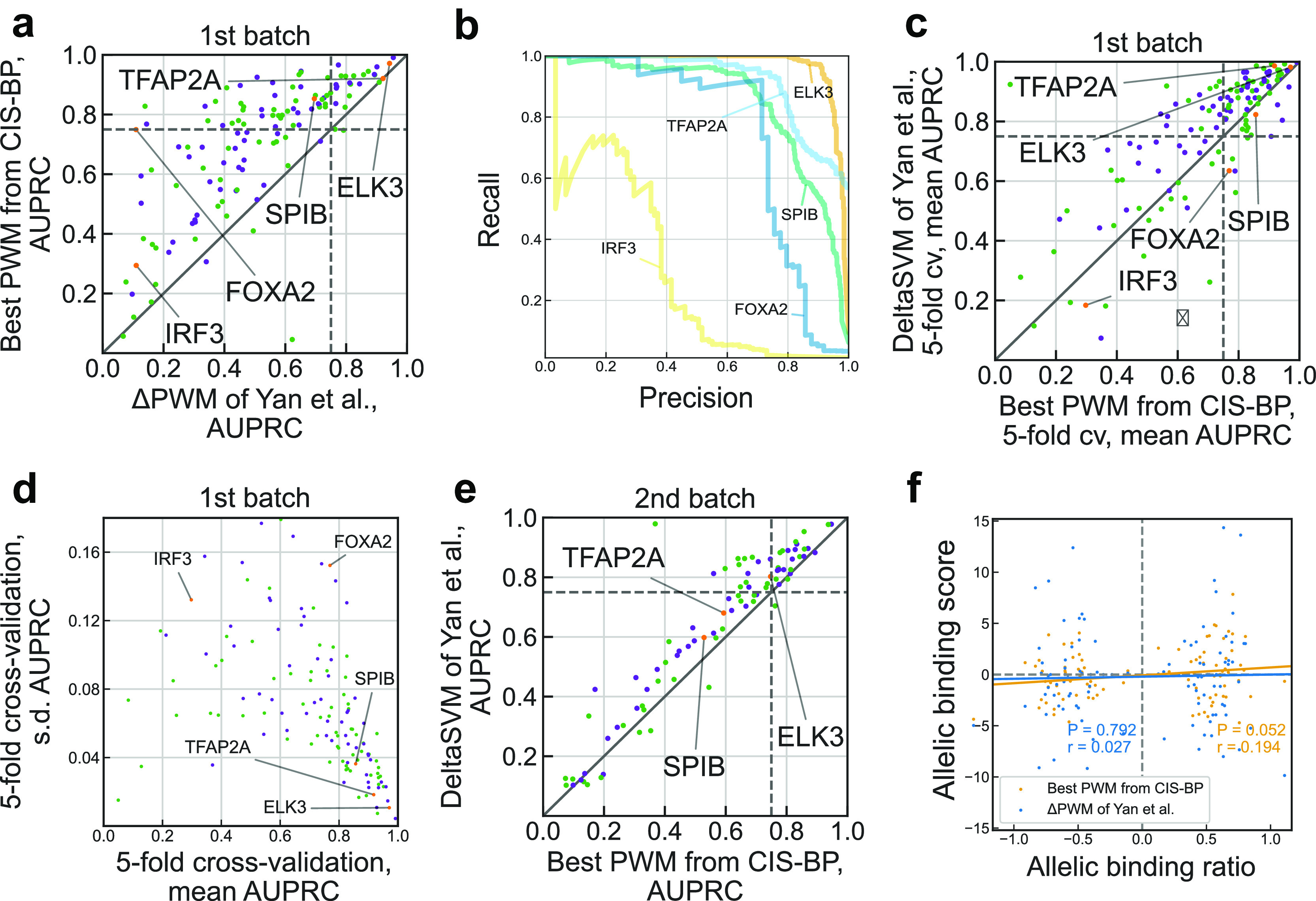
Re-evaluation of position weight matrices with the SNP-SELEX data. a. Comparison of performance of Yan *et al*. ΔPWM (x-axis) and best CIS-BP position weight matrices (PWMs) in predicting preferential binding SNPs in the 1st batch on the SNP-SELEX data. Each point denotes one of 129 TFs, violet and green points denote inferred and direct PWMs, respectively (see the Methods). Both axes show area under the precision-recall curve (AUPRC) values. Transcription factors (TFs) shown in Figure 2b of Yan *et al*. are highlighted in orange and labeled. Dashed lines denote AUPRC of 0.75. b. Examples of the precision-recall curves showing performance of different PWM models in predicting preferential binding SNPs (single-nucleotide polymorphisms) as in Figure 2b of Yan *et al*. c. Comparison of performance of deltaSVM (y-axis) and best CIS-BP PWMs (x-axis) in predicting preferential binding SNPs identified in the 1st batch of SNP-SELEX. Each point denotes one of 129 TFs, violet and green points denote inferred and direct PWMs, respectively. Both axes show mean AUPRC values obtained by 5-fold cross-validation (cv). Dashed lines denote AUPRC of 0.75. d. Variance of performance of CIS-BP PWMs (x-axis: mean AUPRC, y-axis: s.d.) in 5-fold cross-validation using the complete data of the 1st batch of SNP-SELEX. Each point denotes one of 129 TFs, violet and green points denote inferred and direct PWMs, respectively. e. Comparison of performance of deltaSVM (y-axis) and best CIS-BP PWMs (x-axis) in predicting preferential binding SNPs identified in the 2nd batch of SNP-SELEX. Each point denotes one of 87 TFs, violet and green points denote inferred and direct PWMs, respectively. Both axes show AUPRC values. Dashed lines denote AUPRC of 0.75. f. Correlation of allelic biases of DNA binding detected from ChIP-Seq experiments in HepG2 cells by Yan *et al*. and those predicted by ΔPWM of Yan *et al*. (blue) and best CIS-BP PWMs (orange). Pearson correlation coefficient ( *r*) and the respective *P*-value are shown. The allelic binding ratio is computed as in Yan *et al*.; 101 transcription factor-SNP pairs involving 68 unique SNPs and 6 transcription factors (ATF2, FOXA2, HLF, MAFG, YBX1, and FOXA1) are shown.

Next, we compared the overall performance of ΔPWM ^CIS-BP^ for different TFs at the 1st SNP-SELEX batch. For more than a half (72 of 129) of transcription factors ΔPWM ^CIS-BP^ achieved reliable predictions fulfilling the same criterion as in Yan *et al*. of the AUPRC > 0.75, see Figure 2a. This is 3 times more transcription factors with reliable PWM predictions than reported in Yan *et al*. for ΔPWM ^Mult^ (only 24 out of 129). Notably, we obtained good predictions in some cases reported as markedly underperforming such as FOXA2 (compare Figure 2b with Figure 2b of Yan *et al*.). Another TF performing markedly poorly for PWM ^Mult^ was IRF3, but the best PWM ^CIS-BP^ performed better than both ΔPWM ^Mult^ and deltaSVM (AUPRC for ΔPWM ^CIS-BP^ of 0.298 as compared to 0.184 of deltaSVM). In some cases, the predictive power of ΔPWM ^CIS-BP^ went in line with that of ΔPWM ^Mult^, for instance, TFAP transcription factors in both cases displayed outstanding performance (AUPRC of 0.9 for ΔPWM ^Mult^ and 0.92 for ΔPWM ^CIS-BP^) whereas E2F family transcription factors in both cases performed worse (AUPRC of 0.4 for ΔPWM ^Mult^ and 0.42 for ΔPWM ^CIS-BP^).

In fact, for 34 transcription factors, PWM ^CIS-BP^ outperformed advanced models of deltaSVM ( Figure 2c). 5-fold cross-validation showed that models reaching higher AUPRC simultaneously had a lower variance in prediction quality across individual folds ( Figure 2d). Furthermore, we tested the PWMs on the independent 2nd batch data ( Figure 2e, compare with Figure 3d of Yan *et al*.), and it also showed competitive albeit lower performance, with 36 of 124 transcription factors passing 0.75 AUPRC. Finally, we tested if the PWM predictions agreed with the allelic binding ratios in HepG2 ChIP-seq data and found a small but marginally significant correlation ( Figure 2f, *r* = 0.194, *P* = 0.052) for 101 SNPs tested in Yan *et al*. and reaching *r* = 0.235 ( *P* = 0.047) for a subset of 72 SNPs with significant PWM ^CIS-BP^ hits (motif *P*-value < 0.005), in contrast to almost zero correlation for ΔPWM ^Mult^ reported in Yan *et al*.

## Discussion

Our approach mimics a machine learning setup, where the best model is selected ("trained") through cross-validation on a first experimental data set (1st batch of the SNP-SELEX data), and then additionally independently validated on the second experimental data set (2nd batch of SNP-SELEX data). As we select from a finite and typically small set of candidate PWMs, the risks of overfitting are minimized, and the resulting performance was not correlated with the number of ‘candidate’ PWMs. The utilized layout allowed us to pick up the best suited PWM independently from the original data or motif discovery method used for PWM construction, yet maintaining the main PWM limitations, such as the assumption of the independent contributions of nucleotides at different TFBS positions.

The lower performance of PWMs for TFBS recognition as compared with more complex models was reported in many publications. ^12^ ^–^ ^14^ The popular explanation blames the assumption of positional independence of PWM scores, which comes short of taking into account the marked correlations of nucleotides located at neighboring or even distant positions of binding sites. ^15^ ^–^ ^17^ This shortcoming is also considered over-restrictive for PWM applications in predicting the effects of single nucleotide variants on TF binding, ^4^ ^,^ ^18^ which has recently come into the spotlight of modern genetics where the advent of complete genome sequencing brought about the need for interpretation of phenotypes associated with regulatory variants. ^19^ ^,^ ^20^ Here we suggest an alternative explanation of inadequate PWM performance in predicting the effects of single-nucleotide variants on TF binding. In many cases, the reason is an inadequate PWM construction or selection procedure.

Besides technical difficulties in proper “training” a PWM through motif discovery from different types of experimental data, the particular experimental context may influence the applicability of the resulting PWM. Careful selection of PWMs from a pool of alternative existing models results in an apparent improvement of the quantitative assessment of preferential binding to single-nucleotide variants, especially for high-scoring TFBS. In many cases, the prediction power becomes comparable to that of the significantly more complex model such as deltaSVM. We specifically emphasize that in our study all selected PWM ^CIS-BP^ were genuine PWMs following the classic assumption of the independent contribution of positional scores.

Summing up, our results do not compromise the high performance of deltaSVM, ^12^ used by Yan *et al*. as an advanced substitution of position weight matrices (PWMs). However, properly selected PWMs achieve performance that is very close and in some cases even better than that of deltaSVM. Despite the simplicity of the PWM model, its construction is not trivial and its success depends both on the motif discovery algorithm and reliability of the training data. In our case, almost half of the best PWMs were derived from related TFs, including 8 cases of PWMs based on experimental data from other species. The experiments used to obtain the best PWMs were also of different types, including ChIP-Seq, protein-binding microarrays, and SMiLE-Seq data, see *Extended data,* Supplementary Table S1. ^21^ Thus, it is important to consider various sources of PWMs and select those the most suitable by proper benchmarking. In the context of applying PWMs to analyze regulatory variants, SNP-SELEX of Yan *et al*. provides rich, unique, and practically useful data.

The objective of our study is by no means to undermine the necessity of complex TFBS models with dependent positional contributions. Advanced multiparametric and alignment-free approaches such as deltaSVM appear very likely to shape the oncoming future of transcription factor binding site models. Rather, we want to underline that the prediction performance of transcription factor binding sites in its current stage is more influenced by model training protocols than by model structure restrictions. PWMs still can deliver a solid standard in representation and bioinformatics analysis of the transcription factor binding sites, including assessment of the functional impact of single nucleotide variants in gene regulatory regions. In addition, we underline that better defined ‘baseline’ PWMs or PWM selection procedures are required for the proper evaluation of advanced models. It is important that such ‘baseline’ TFBS models, while certainly being handicapped by design, still reach meaningful prediction quality. These are good news for thousands of researchers who still use the ‘legacy’ PWM scoring for practical applications in regulatory genomics and bioinformatics.

## Methods

### PWMs used in the study

As a source of candidate PWMs we used the CIS-BP (Catalog of Inferred Sequence Binding Preferences) collection ^9^ ^,^ ^10^ of pre-made matrices. For each TF, we gathered all PWMs assigned to the TF and added PWMs for related proteins sharing similar DNA binding domains. This was motivated by the results of the benchmarking study of Ambrosini *et al*. ^2^ where a PWM for some TF often displayed poorer TFBS recognition power than a PWM for some different TF but with the same DNA-binding domains.

The starting set of position frequency matrices was extracted from TF_Information_all_motifs.txt of CIS-BP 2.0 that includes models derived from direct experimental data for each TF and models that can be inferred given the TF family-specific threshold on DNA-binding domain similarity, see Ref. 11. In Figure 2 such PWMs are referred to as ‘direct’ and ‘inferred’. All position frequency matrices were converted to log-odds PWMs as in Ref. 22 with an arbitrarily selected word count of 100, a pseudocount of 1, and uniform background nucleotide probabilities. For each TF, the set of PWMs was additionally extended by considering related TFs, i.e. PWMs for all ETV* TFs were added to the ETV1 PWM set, all FOX* (Forkhead box) PWMs were added to the FOXA2 PWM set, etc. (e.g. YY1 and YY2 PWM sets were identical). This procedure was not performed for ZNF* (zinc finger) TFs as these TFs can recognize very dissimilar motifs and thus additional PWMs of other ZNFs would unlikely provide any benefit.

### Determination of transcription factor binding preference using PWMs

To assess with a particular PWM whether an SNV affects transcription factor binding, we used PERFECTOS-APE ^6^ that estimates the log-fold change of motif *P*-values computed for best PWM hits detected among sites overlapping the first and the second of two alternative alleles. To use the prediction as a binary classifier, we treated the cases with *P* > 0.005 at both alleles as predicted negatives and used the log-fold change as the prediction score in the remaining cases. The auc function of the sklearn.metrics Python package was used to estimate the area under the precision-recall curve (AUPRC).

### Estimating PWM performance with SNP-SELEX data

To provide a fair assessment, we mimicked the benchmarking protocol of Yan *et al*. Particularly, true positives and true negatives were selected from the SNP-SELEX data as follows. 1st batch data positives: PBS *P*-value < 0.01 and OBS *P*-value < 0.05; negatives: PBS *P*-value > 0.5 and OBS *P*-value < 0.05. 2nd batch data positives: PBS *P*-value < 0.01, negatives: PBS *P*-value > 0.5. For each TF, we tested each CIS-BP PWM from its PWM set. For each TF, the PWM reaching the highest AUPRC on the 1st batch data was selected for evaluation against the best PWM on the 1st batch ( Figure 2a) and against deltaSVM on the 2nd batch of SNP-SELEX data ( Figure 2e). Performance estimates for deltaSVM models (used in Figure 2c,e) were extracted from Supplementary Table S7 of Yan *et al*. Performance estimates of ΔPWM ^Mult^ (used in Figure 2a) were kindly shared on our request by the authors. ^4^ We also mimicked the stratified five-fold cross-validation procedure used by Yan *et al.* The mean of AUPRC across the folds was used to compare the performance of ΔPWM ^CIS-BP^ with deltaSVM of Yan *et al.* at the first batch of SNP-SELEX data ( Figure 2c).

### Applying PWMs for analysis of allele-specific binding

The data on allelic binding ratios at individual SNPs and respective ΔPWM predictions of Yan *et al*. ( Figure 2f, compare to Figure 2d of Yan *et al*.) were kindly shared on our request by the authors. The data included 193 TF-SNP pairs demonstrating allelic imbalance with 101 of 193 pairs annotated with the ΔPWM predictions. For these SNPs, we obtained PWM predictions with the same protocol as for the SNP-SELEX data using the best PWMs selected with the 1st batch of the SNP-SELEX data.

## Data availability

### Source data

Original data on preferential binding SNPs as well as ΔPWM and deltaSVM predictions are provided in the supplementary materials section of the Yan *et al*. paper. ^4^

CISBP Human PWMs collection was extracted from CIS-BP 2.0. ^9^ ^,^ ^10^

### Extended data

Figshare: PWM-evaluation-using-SNP-SELEX, https://doi.org/10.6084/m9.figshare.16906789.v1. ^21^

This project contains the following extended data:

• **Supplementary table S1** (Overview of PWMs and their performance in recognizing SNPs affecting transcription factor binding in SNP-SELEX data.)
• **Supplementary figure S1** (Performance of ΔPWM ^CIS-BP^ in predicting weak and strong TF binding sites.)

Data are available under the terms of the Creative Commons Zero “No rights reserved” data waiver (CC0 1.0 Public domain dedication).

## References

[1] Wasserman WW Sandelin A : Applied bioinformatics for the identification of regulatory elements. *Nat. Rev. Genet.* 2004;5:276–287. 10.1038/nrg1315 15131651

[2] Ambrosini G : Insights gained from a comprehensive all-against-all transcription factor binding motif benchmarking study. *Genome Biol.* 2020;21:114. 10.1186/s13059-020-01996-3 32393327 PMC7212583

[3] Kibet CK Machanick P : Transcription factor motif quality assessment requires systematic comparative analysis. *F1000Research.* 2015;4(ISCB Comm J):1429. 10.12688/f1000research.7408.2 PMC4821295 27092243

[4] Yan J : Systematic analysis of binding of transcription factors to noncoding variants. *Nature* 2021;591:147–151. 10.1038/s41586-021-03211-0 33505025 PMC9367673

[5] Macintyre G Bailey J Haviv I : is-rSNP: a novel technique for in silico regulatory SNP detection. *Bioinformatics* 2010;26:i524–i530. 10.1093/bioinformatics/btq378 20823317 PMC2935445

[6] Vorontsov IE Kulakovskiy IV Khimulya G : PERFECTOS-APE - Predicting Regulatory Functional Effect of SNPs by Approximate P-value Estimation. *Proceedings of the International Conference on Bioinformatics Models, Methods and Algorithms 102–108 (SCITEPRESS - Science and and Technology Publications* 2015. 10.5220/0005189301020108

[7] Coetzee SG Coetzee GA Hazelett DJ : motifbreakR: an R/Bioconductor package for predicting variant effects at transcription factor binding sites. *Bioinformatics* 2015;31:btv470–bt3849. 10.1093/bioinformatics/btv470 26272984 PMC4653394

[8] Deplancke B Alpern D Gardeux V : The Genetics of Transcription Factor DNA Binding Variation. *Cell* 2016;166:538–554. 10.1016/j.cell.2016.07.012 27471964

[9] Lambert SA : The Human Transcription Factors. *Cell* 2018;172:650–665. 10.1016/j.cell.2018.01.029 29425488 PMC12908702

[10] Weirauch MT : Determination and Inference of Eukaryotic Transcription Factor Sequence Specificity. *Cell* 2014;158:1431–1443. 10.1016/j.cell.2014.08.009 25215497 PMC4163041

[11] Yin Y : Impact of cytosine methylation on DNA binding specificities of human transcription factors. *Science* 2017;356:eaaj2239. 10.1126/science.aaj2239 28473536 PMC8009048

[12] Grau J Posch S Grosse I : A general approach for discriminative de novo motif discovery from high-throughput data. *Nucleic Acids Res.* 2013;41(21):e197. 10.1093/nar/gkt831 24057214 PMC3834837

[13] Siebert M Söding J : Bayesian Markov models consistently outperform PWMs at predicting motifs in nucleotide sequences. *Nucleic Acids Res.* 2016;44(13):6055–6069. 10.1093/nar/gkw521 27288444 PMC5291271

[14] Guo Y Tian K Zeng H : A novel k-mer set memory (KSM) motif representation improves regulatory variant prediction. *Genome Res.* 2018;28(6):891–900. 10.1101/gr.226852.117 29654070 PMC5991515

[15] Mordelet F Horton J Hartemink AJ : Stability selection for regression-based models of transcription factor-DNA binding specificity. *Bioinformatics (Oxford, England).* 2013;29(13):i117–i125. 10.1093/bioinformatics/btt221 23812975 PMC3694650

[16] Le DD Shimko TC Aditham AK Comprehensive, high-resolution binding energy landscapes reveal context dependencies of transcription factor binding. *Proc. Natl. Acad. Sci. U. S. A.* 2018;115(16):E3702–E3711. 10.1073/pnas.1715888115 29588420 PMC5910820

[17] Dresch JM Zellers RG Bork DK : Nucleotide Interdependency in Transcription Factor Binding Sites in the Drosophila Genome. *Gene Regul. Syst. Biol.* 2016;10:21–33. 10.4137/GRSB.S38462 27330274 PMC4907338

[18] Lee D : A method to predict the impact of regulatory variants from DNA sequence. *Nat. Genet.* 2015;47:955–961. 10.1038/ng.3331 26075791 PMC4520745

[19] Degtyareva AO Antontseva EV& Merkulova TI : Regulatory SNPs: Altered Transcription Factor Binding Sites Implicated in Complex Traits and Diseases. *Int. J. Mol. Sci.* 2021:22(12):6454. 10.3390/ijms22126454 34208629 PMC8235176

[20] Huo Y Li S Liu J : Functional genomics reveal gene regulatory mechanisms underlying schizophrenia risk. *Nat. Commun.* 2019;10(1):670. 10.1038/s41467-019-08666-4 30737407 PMC6368563

[21] Abramov S : PWM evaluation using SNP-SELEX. figshare. 10.6084/m9.figshare.16906789.v1

[22] Lifanov AP Makeev VJ Nazina AG : Homotypic Regulatory Clusters in Drosophila. *Genome Res.* 2003;13:579–588. 10.1101/gr.668403 12670999 PMC430164

