# Supplementary material for: Positional weight matrices have sufficient prediction power for analysis of noncoding variants
Source: F1000Res. 2022 Jul 4;11:33. Originally published 2022 Jan 12. [Version 3] doi: 10.12688/f1000research.75471.3 (PMC9237556; doi:10.12688/f1000research.75471.3)
Supplement: Peer review history

# Peer review: Reviewer response for version 2

**DOI:** 10.5256/f1000research.135316.r141836

**Published:** 2022-06-27

**Authors:** Philip Machanick

**Affiliations:** 1 Computer Science, Rhodes University, Makhanda, South Africa

Thank you for the updates.

 They are a sufficient response to my initial review.

 I include citation of one of my papers ^1^ as further reading. Motif assessment is a difficult topic and there is no one solution that fits all cases.

Are arguments sufficiently supported by evidence from the published literature or by new data and results?

Partly

Is the conclusion balanced and justified on the basis of the presented arguments?

Partly

Is the rationale for commenting on the previous publication clearly described?

Partly

Are any opinions stated well-argued, clear and cogent?

Partly

Reviewer Expertise:

Transcription factor binding specificity (computer science perspective).

I confirm that I have read this submission and believe that I have an appropriate level of expertise to confirm that it is of an acceptable scientific standard.

## References

[1] : Transcription factor motif quality assessment requires systematic comparative analysis. *F1000Res* .2015;4: 10.12688/f1000research.7408.2 10.12688/f1000research.7408.2 27092243PMC4821295

# Peer review: Reviewer response for version 2

**DOI:** 10.5256/f1000research.135316.r141837

**Published:** 2022-06-27

**Authors:** Victor G. Levitsky

**Affiliations:** 1 Department of System Biology, Institute of Cytology and Genetics, Novosibirsk, Russian Federation

Though I still disagree with authors on the principal motivation of study, i.e. I mean that the traditional PWM and the alternative models neither good or bad, they predict sites of different structure. But let the current paper reflects the advantages of PWMs, so I agree that the manuscript could be accepted

Are arguments sufficiently supported by evidence from the published literature or by new data and results?

Partly

Is the conclusion balanced and justified on the basis of the presented arguments?

Partly

Is the rationale for commenting on the previous publication clearly described?

Partly

Are any opinions stated well-argued, clear and cogent?

No

Reviewer Expertise:

bioinformatics, massive analysis of genome data

I confirm that I have read this submission and believe that I have an appropriate level of expertise to confirm that it is of an acceptable scientific standard.

# Peer review: Reviewer response for version 1

**DOI:** 10.5256/f1000research.79349.r128877

**Published:** 2022-04-07

**Authors:** Philip Machanick

**Affiliations:** 1 Computer Science, Rhodes University, Makhanda, South Africa

The Yan *et al*. article is a useful addition to the literature so questioning the validity of their results is also useful. However, this article makes an exaggerated claim of the extent to which Yan *et al.* reduce the utility of PWMs.

 PWMs are already known to be potentially flawed models, varying from very accurately predicting DNA binding specificity to poorly doing so, with potential confounders like cofactors ^1^ and indirect binding ^3^. The extent to which these issues apply can depend on the method used to determine the PWM. For example, using ChIP-seq data can create a composite motif incorporating part of a cofactor. Using PBM may eliminate this effect, but can produce poor binding specificity in some cases, possibly because either the specificity is mediated by cofactor requirements or because binding is indirect ^4^^,^^5^.

 While I am specifically mentioning older approaches like PBMs here, any *in vitro* or *in silico* method potentially has similar issues.

 For this reason, I advocate using a range of methods to assess motif quality ^2^.

 As regards SNPs and other variability, this sort of issue has to be taken into account, otherwise any variation in specificity may not correspond to *in vivo* reality.

 So, back to the approach of this paper: selecting PWMs that match specific criteria for reliability. It is not clear to me that this in any way invalidates the results of Yan *et al*. as there is variability in the predictive quality of PWMs, given the potential for confounders.

 I would like to see a clearer explanation of the extent to which Yan *et al*. actually diminish the utility of PWMs (noting this is in a specific context, assessing small genomic variants) and the extent to which this review generalises beyond carefully selected PWMs..

Are arguments sufficiently supported by evidence from the published literature or by new data and results?

Partly

Is the conclusion balanced and justified on the basis of the presented arguments?

Partly

Is the rationale for commenting on the previous publication clearly described?

Partly

Are any opinions stated well-argued, clear and cogent?

Partly

Reviewer Expertise:

Transcription factor binding specificity (computer science perspective).

I confirm that I have read this submission and believe that I have an appropriate level of expertise to confirm that it is of an acceptable scientific standard, however I have significant reservations, as outlined above.

## References

[1] : A structure-based Multiple-Instance Learning approach to predicting in vitro transcription factor-DNA interaction. *BMC Genomics* .2015;16 Suppl 4: 10.1186/1471-2164-16-S4-S3 S3 10.1186/1471-2164-16-S4-S3 25917392PMC4416172

[2] : Transcription factor motif quality assessment requires systematic comparative analysis. *F1000Res* .2015;4: 10.12688/f1000research.7408.2 10.12688/f1000research.7408.2 27092243PMC4821295

[3] : Insights gained from a comprehensive all-against-all transcription factor binding motif benchmarking study. *Genome Biology* .2020;21(1) : 10.1186/s13059-020-01996-3 10.1186/s13059-020-01996-3 32393327PMC7212583

[4] : Protein-binding microarray analysis of tumor suppressor AP2α target gene specificity. *PLoS One* .2011;6(8) : 10.1371/journal.pone.0022895 e22895 10.1371/journal.pone.0022895 21876733PMC3158074

[5] : Non-DNA-binding cofactors enhance DNA-binding specificity of a transcriptional regulatory complex. *Mol Syst Biol* .2011;7: 10.1038/msb.2011.89 555 10.1038/msb.2011.89 22146299PMC3737730

# Response

**Published:** 2022-06-21

**Authors:** Sergey Abramov

**Affiliations:** Vavilov Institute of General Genetics, Russian Federation

**The Yan et al. article is a useful addition to the literature so questioning the validity of their results is also useful. However, this article makes an exaggerated claim of the extent to which Yan et al. reduce the utility of PWMs.**

 We did our best to clarify our claim as it was also questioned by Dr. Levitsky.

**PWMs are already known to be potentially flawed models, varying from very accurately predicting DNA binding specificity to poorly doing so, with potential confounders like cofactors 1 and indirect binding 3. The extent to which these issues apply can depend on the method used to determine the PWM. For example, using ChIP-seq data can create a composite motif incorporating part of a cofactor. Using PBM may eliminate this effect, but can produce poor binding specificity in some cases, possibly because either the specificity is mediated by cofactor requirements or because binding is indirect 4,5.**

 We fully agree that the data source and the computational procedure used to derive the TFBS model would significantly affect the result in terms of whether it reflects the genuine TF binding specificity or significantly depends on confounding factors. In this paper we restricted ourselves to a more specific context of using PWMs for quantifying the variants identified with the SNP-SELEX, which is an in vitro assay, so indirect binding and cofactors do not influence the outcome. To make it clear, we have revised the Introduction section of our manuscript. 

**For this reason, I advocate using a range of methods to assess motif quality 2.**

 Indeed. A comprehensive assessment of motif models using different types of experimental data was performed e.g. in Ambrosini et al. 2022. In this study, we did not focus on selecting the optimal PWMs for a wide range of practical applications or in terms of representing in vivo binding. Our aim was to demonstrate that PWMs provide the type of a model, which is able to show a reasonable performance in classifying differentially bound oligonucleotides with single-nucleotide substitutions.

**So, back to the approach of this paper: selecting PWMs that match specific criteria for reliability. It is not clear to me that this in any way invalidates the results of Yan et al. as there is variability in the predictive quality of PWMs, given the potential for confounders. I would like to see a clearer explanation of the extent to which Yan et al. actually diminish the utility of PWMs (noting this is in a specific context, assessing small genomic variants) and the extent to which this review generalises beyond carefully selected PWMs.**

 The same issue was pointed out by Dr. Levistky and we did our best to clarify the aim of the study in the revised version of the manuscript. We believe, that quantifying the effects of single nucleotide variants on TF binding is an important practical problem emerging in the increasingly influential field of personalized genomics, as according to the recent reports up to 80% of causal variants are found in the regulatory regions [see e.g. https://www.medrxiv.org/content/10.1101/2021.06.08.21258515v2]. Even though this is a limited problem, it is worth clarifying the PWM performance for this particular application. We have added the necessary information in the Introduction section.

# Peer review: Reviewer response for version 1

**DOI:** 10.5256/f1000research.79349.r123085

**Published:** 2022-02-14

**Authors:** Victor G. Levitsky

**Affiliations:** 1 Department of System Biology, Institute of Cytology and Genetics, Novosibirsk, Russian Federation

1.

 Boytsov et al. in the Abstract of their correspondence cited Yan et al. paper

 Yet, recently Yan et al. presented new experimental method for analysis of regulatory variants and, based on its results, reported that "the position weight matrices of most transcription factors lack sufficient predictive power". Here, we reanalyze the rich experimental dataset obtained by Yan et al. and show that appropriately selected position weight matrices in fact can successfully quantify transcription factor binding to alternative alleles…

 But actually, Yan et al. in the Abstract wrote:

 …the position weight matrices of most transcription factors lack sufficient predictive power, whereas the support vector machine combined with the gapped k-mer representation show much improved performance, when assessed on results from independent SNP-SELEX experiments involving a new set of 61,020 sequence variants….

 I think that Yan et al. are not wrong

 Since, in particular, Yan et al. also wrote that

 …(1) We reasoned that the poor performance of many PWMs was probably because they did not take into account dinucleotide interdependency in transcription factor–DNA interactions and the influence of flanking DNA sequences11,12. Previous studies have shown that dinucleotide interdependency exists for some transcription factor dimers4. For example, according to the PWM model, the SNP rs79124498—located within a binding site of HLF, a bZIP family transcription factor that binds DNA as homodimers—would have little effect on HLF binding. However, SNP-SELEX indicated that the G allele bound more strongly than the T allele to HLF. This could be caused by the dinucleotide interdependency between position 2 (the SNP position) and position 10 in the binding site (Fisher’s exact test P < 2.2 × 10−16, odds ratio = 3.34)…

 …(2) PWM performed poorly for SNPs located in low-affinity binding sites of transcription factors. However, this limitation could be overcome by using deltaSVM. When we categorized SNPs into five quantiles on the basis of their binding affinities as measured by OBS, and assessed the performance of PWM and deltaSVM in predicting their allelic binding by fivefold cross-validation or using the novel batch of SNP-SELEX experimental results (Extended Data Fig. 7), deltaSVM outperformed PWM in all quantiles, particularly in the lower quantiles corresponding to weak transcription factor binding sites…..

 Does Boytsov et al. not agree with Yan et al. in these two points?

 As I know, the alternative model can outperform the standard PWM model (e.g. BaMM, Siebert,M. and Söding,J. 2016 Bayesian Markov models consistently outperform PWMs at predicting motifs in nucleotide sequences. Nucleic Acids Res., 44, 6055–6069). At least this should be if an alternative model incorporates a PWM model (as BaMM does).

 Hence, Yan et al. compared PWM (e.g. in BEESEM realization) and deltaSVM, and proved that PWMs are worse than deltaSVM. Boytsov et al. used additional PWMs from public databases such as CIS-BP to select the best performed PWM. This is actually proves that ready PWMs (that respects to the same family, i.e. to other TFs with the same DNA binding domain) may be quite successive, but this does not prove that PWMs are better than deltaSVMs. This also does not imply that PWMs are good or bad. We should develop a special pipeline to compare PWMs and deltaSVMs (this is out of scope of paper). Boytsov et al. did not tried to incorporate the non-traditional model deltaSVMs and data from CIS-BP to potentiate the performance of deltaSVMs. So about what the Boytsov paper? Hence, Boytsov et al. proved that BEESEM realization may be better if we incorporate CIS-BP data or what?

 Boytsov et al. concluded

 …However, properly selected PWMs achieve performance that is very close and in some cases even better than that of deltaSVM. Despite the simplicity of the PWM model, its construction is not trivial and its success depends both on the motif discovery algorithm and reliability of the training data…

 Any motif discovery algorithm does not use any motif library on the process of de novo search. Usually, motif libraries are applied to interpret enriched motifs (e.g. STREME and Tomtom in meme suite, https://meme-suite.org/meme/index.html) Hence, application of motifs library is not a step in de novo process. At this step, I again does not understand why Boytsov et al. compared Figure 1b with Fig. 2b of Yan et al.

 Overall, Boytsov et al. should draw attention to the point of disagreement with data or conclusion of Yan et al. paper.

 2.

 The TF classification by family is wrongly described.

 …For each TF, the set of PWMs was additionally extended by considering related TFs, i.e. PWMs for all ETV* TFs were added to the ETV1 PWM set, all FOX* (Forkhead box) PWMs were added to the FOXA2 PWM set, etc. (e.g. YY1 and YY2 PWM sets were identical). This procedure was not performed for ZNF* (zinc finger) TFs as these TFs can recognize very dissimilar motifs and thus additional PWMs of other ZNFs would unlikely provide any benefit…

 This description does not explain several pair from Supplementary Data (Overview of best CIS-BP PWMs), e.g.ETV2 & FLI1

 The correct and default approach was described in the previous publication (Ambrosini G , et al. Genome Biol. 2020: Matrices were manually mapped to gene symbols and TF families from TFclass [Wingender E,et al.. Nucleic Acids Res. 2018] and CIS-BP). Moreover, the CIS-BP database contains TF PWMs that were already classified by families.

 3.

 Currently, links to Figure 1 are contained in the Introduction section. Although the format of correspondence paper is flexible, I propose that authors should either do not user various sections, or apply the standard sections, Introduction, Methods, Results, Conclusions/Discussion.

Are arguments sufficiently supported by evidence from the published literature or by new data and results?

Partly

Is the conclusion balanced and justified on the basis of the presented arguments?

Partly

Is the rationale for commenting on the previous publication clearly described?

Partly

Are any opinions stated well-argued, clear and cogent?

No

Reviewer Expertise:

bioinformatics, massive analysis of genome data

I confirm that I have read this submission and believe that I have an appropriate level of expertise to confirm that it is of an acceptable scientific standard, however I have significant reservations, as outlined above.

## References

[1] : Systematic analysis of binding of transcription factors to noncoding variants. *Nature* .591(7848) : 10.1038/s41586-021-03211-0 147-151 10.1038/s41586-021-03211-0 33505025PMC9367673

[2] : Bayesian Markov models consistently outperform PWMs at predicting motifs in nucleotide sequences. *Nucleic Acids Research* .2016;44(13) : 10.1093/nar/gkw521 6055-6069 10.1093/nar/gkw521 PMC529127127288444

[3] : Insights gained from a comprehensive all-against-all transcription factor binding motif benchmarking study. *Genome Biology* .2020;21(1) : 10.1186/s13059-020-01996-3 10.1186/s13059-020-01996-3 32393327PMC7212583

# Response

**Published:** 2022-06-21

**Authors:** Sergey Abramov

**Affiliations:** Vavilov Institute of General Genetics, Russian Federation

**… But actually, Yan et al. in the Abstract wrote: …the position weight matrices of most transcription factors lack sufficient predictive power, whereas the support vector machine combined with the gapped k-mer representation show much improved performance, when assessed on results from independent SNP-SELEX experiments involving a new set of 61,020 sequence variants….**

**I think that Yan et al. are not wrong…**

 In fact, we do not challenge the authors' statement regarding the high performance of deltaSVM in predicting the SNP-SELEX results by sequence analysis. Particularly, we explicitly state that 

*"...  our results do not compromise the high performance of deltaSVM, used by Yan et al. as an advanced substitution of position weight matrices (PWMs)" (paragraph 4 of Discussion).*

 Yet, we strongly disagree with the authors' conclusion on the PWM performance ("lack sufficient predictive power") and believe that their comparison of the performance of the PWM and deltaSVM became one-sided in favor of deltaSVM due to an accidental selection of particular PWMs in their study. We believe that public databases contain PWM which can display much better performance in quantifying allele-specific binding and put it explicitly in the Introduction that 

*“We show that the careful selection of PWMs of many TFs from a public database quantitatively explains the differential TF binding to allelic variants with reliability comparable to that of deltaSVM.”*

 Again, we put into Discussion that

*“Summing up, our results do not compromise the high performance of deltaSVM, used by Yan et al. as an advanced substitution of position weight matrices (PWMs). However, properly selected PWMs achieve performance that is very close and in some cases even better than that of deltaSVM.”* 

 We have also added a detailed discussion on the subject into the Discussion section (see the response to the next question of the reviewer).

**Since, in particular, Yan et al. also wrote that …(1) We reasoned that the poor performance of many PWMs was probably because they did not take into account dinucleotide interdependency in transcription factor–DNA interactions and the influence of flanking DNA sequences…**

**…(2) PWM performed poorly for SNPs located in low-affinity binding sites of transcription factors.**

**Does Boytsov et al. not agree with Yan et al. in these two points?**

 We agree with the theoretical limitations of position weight matrices regarding their inability to account for non-additive contributions of particular nucleotides into protein affinities. Yet, it is important to distinguish between the general and well-known limitations of the PWM as a model and the low performance of particular matrices. In our opinion the poor performance of PWMs used in the study of  Yan et. al. was not due to the intrinsic inability of PWMs to classify TF binding preferences at particular TFBS, but rather due to the way the PWMs used in this study were selected/constructed. We prove it by providing the alternative PWMs that belong to the same class of mononucleotide models but perform better than the PWMs of Yan et al. and comparably to deltaSVM. We have added an explicit statement on this matter in the revised version of the manuscript.

*“The objective of our study is by no means to undermine the necessity of complex TFBS models with dependent positional contributions. Advanced multiparametric and alignment-free approaches such as deltaSVM appear very likely to shape the oncoming future of transcription factor binding site models. Rather, we want to underline that the prediction performance for transcription factor binding sites in its current stage is more influenced by model training protocols than by model structure restrictions. PWMs still can deliver a solid standard in representation and bioinformatics analysis of the transcription factor binding sites, including assessment of the functional impact of single nucleotide variants in gene regulatory regions. In addition, we underline that better defined 'baseline' PWMs or PWM selection procedures are required for the proper evaluation of advanced models. It is important that such 'baseline' TFBS models, while certainly being handicapped by design, still reach meaningful prediction quality.”*

**… ready PWMs (that respects to the same family, i.e. to other TFs with the same DNA binding domain) may be quite successive, but this does not prove that PWMs are better than deltaSVMs.**

 We used PWMs with the same DNA binding domains to increase the repertoire of candidate PWMs, from which the best PWM for assessing variants identified by SNP-SELEX experiments can be selected. To avoid confusion we have added two subsections to the Methods section: “PWMs used in the study” and “Selection of the best PWM for a TF”. In fact, carefully selected PWMs outperformed deltaSVM models for 34 of 129 TFs (see paragraph 3 of Introduction in the manuscript and Figure 2), and many of these PWMs were initially constructed for different TFs and even different species (see Supplementary Table S1). This does not compromise better deltaSVM performance for other TFs (see Fig. 1e).

**Hence, Boytsov et al. proved that BEESEM realization may be better if we incorporate CIS-BP data or what?**

 We did not test BEESEM or other types of motif discovery software or alternative PWM-like motif representations, and thus don’t know if they provide even better PWMs than we found in CIS-BP. We only used the existing published PWMs available in the CIS-BP database. SNP-SELEX provides a rich data source to test various types of models in the task of predicting rSNP effects on transcription factor binding, but such testing does not fit the scope of our manuscript.

**Hence, application of motifs library is not a step in de novo process. At this step, I again does not understand why Boytsov et al. compared Figure 1b with Fig. 2b of Yan et al.**

 We did not discuss de novo motif discovery. The idea of our study was to verify whether the inadequate performance of PWMs reported in Yan et al. was related to the type of the model or if it characterized the particular PWMs they used.

 In fact, Yan et al. also did not construct PWMs through de novo motif discovery but used the pre-made PWMs of Yin et al. Similarly, we followed the suit and avoided de novo motif discovery in favor of reusing existing PWMs from CIS-BP. Selection of a single PWM from the pool of related PWMs can be considered as "training" of the model, and we fully replicated the approach of Yan et al. i.e. the cross-validation on the 1st batch of SNP-SELEX data. 

**Overall, Boytsov et al. should draw attention to the point of disagreement with data or conclusion of Yan et al. paper.**

 We did our best to better highlight the key idea of the study in the revised version of the manuscript and added an extensive Discussion section.

**2. The TF classification by family is wrongly described. … This description does not explain several pair from Supplementary Data (Overview of best CIS-BP PWMs), e.g.ETV2 & FLI1**

 In the pool of possible PWMs for each TF, we included CIS-BP 'inferred' PWMs (as described in Methods) which belonged to TFs with a similar DNA-binding domain, hence there is no contradiction. We revised the Methods section, see the subsection “PWMs used in the study.”

**The correct and default approach was described in the previous publication (Ambrosini G , et al. Genome Biol. 2020: Matrices were manually mapped to gene symbols and TF families from TFclass [Wingender E,et al.. Nucleic Acids Res. 2018] and CIS-BP). Moreover, the CIS-BP database contains TF PWMs that were already classified by families.**

 CIS-BP classification of DNA-binding domains is very general and leads to very wide sets of PWMs potentially applicable to a particular TF, if all PWMs across the TF family are considered. To reduce computational complexity, we made a compromise of including 'inferred' motifs (see above) but only for related proteins by matching gene names and not relying on the detailed TF family annotation. Even with this simplification in the PWM selection, which greatly reduced the number of available PWMs, the resulting performance of the best PWMs was significantly better than the PWM performance reported by Yan et al. for the same TF.

 Of note, in Ambrosini et al. (2020) we used all-vs-all testing strategy and reported cross-family applicability of PWMs, although this is computationally ineffective in the practical selection of the best-performing matrices.

**3. Currently, links to Figure 1 are contained in the Introduction section. Although the format of correspondence paper is flexible, I propose that authors should either do not user various sections, or apply the standard sections, Introduction, Methods, Results, Conclusions/Discussion.**

 We have revised the manuscript structure according to the reviewer’s suggestion.
